# Genomic characteristics and evolution of Multicentric Esophageal and gastric Cardiac Cancer

**DOI:** 10.1186/s13062-024-00493-y

**Published:** 2024-07-01

**Authors:** Xi Liu, Lijun Cai, Juan Ji, Dongping Tian, Yi Guo, Shaobin Chen, Meng Zhao, Min Su

**Affiliations:** 1 Institute of Clinical Pathology, Department of Pathology, Shantou University Medical College, Shantou, Guangdong 515041 China; 2 Guangdong Provincial Key Laboratory of Infectious Diseases and Molecular Immunopathology, Shantou University Medical College, Shantou, Guangdong 515041 China; 3 Sichuan Cancer Center, School of Medicine, Sichuan Cancer Hospital & Institute, University of Electronic Science and Technology of China, Chengdu, 610041 China; 4 Cancer Hospital of Shantou University Medical College, Shantou, Guangdong 515041 China; 5 Novogene Co., LTD, Beijing, 100083 China

**Keywords:** Clonality analysis, Sequencing, Multiple cancer, Genomic features

## Abstract

**Background:**

Esophageal carcinoma (EC) and gastric cardiac adenocarcinoma (GCA) have high incidence rates in the Chaoshan region of South China. Multifocal esophageal and cardiac cancer (MECC) is commonly observed in this region in clinical practice. However, the genomic characteristics of MECC remains unclear.

**Materials and methods:**

In this study, a total of 2123 clinical samples of EC and GCA were analyzed to determine the frequency of multifocal tumors, as well as their occurrence sites and pathological types. Cox proportional hazards regression was used to model the relationship between age, sex, and tumor state concerning survival in our analysis of the cohort of 541 patients with available follow-up data. We performed whole-genome sequencing on 20 tumor foci and 10 normal samples from 10 MECC patients to infer clonal structure on 6 MECC patients to explore genome characteristics.

**Result:**

The MECC rate of EC and GCA was 5.65% (121 of 2123). Age and sex were potential factors that may influence the risk of MECC (*p* < 0.001). Furthermore, MECC patients showed worse survival compared with single tumor patients. We found that 12 foci from 6 patients were multicentric origin model (MC), which exhibited significant heterogeneity of variations in paired foci and had an increased number of germline mutations in immune genes compared to metastatic model. In MC cases, different lesions in the same patient were driven by distinct mutation and copy number variation (CNV) events. Although *TP53* and other driver mutation genes have a high frequency in the samples, their mutation sites show significant heterogeneity in paired tumor specimens. On the other hand, CNV genes exhibited higher concordance in paired samples, especially in the amplification of oncogenes and the deletion of tumor suppressor genes.

**Conclusions:**

The extent of inter-tumor heterogeneity suggests both monoclonal and polyclonal origins of MECC, which could provide insight into the genome diversity of MECC and guide clinical implementation.

**Supplementary Information:**

The online version contains supplementary material available at 10.1186/s13062-024-00493-y.

## Background

In the Chaoshan region of South China, esophageal cancer (EC) remains the leading cause of cancer-related death and is frequently accompanied by a high incidence of gastric cardiac adenocarcinoma (GCA). Between 1995 and 2004, the incidence rates of EC and GCA were 74.47 and 34.81 per 100,000 population, respectively [1, 2]. In particular, multifocal esophageal and cardiac cancer (MECC) is a phenomenon commonly observed in this area, where multiple disconnected tumor foci often appear in partially resected samples of the upper gastrointestinal tract. Despite the increasing incidence of multiple primary tumors due to improved diagnostic techniques, clinicians have insufficient understanding and awareness of MECC, which could lead to misdiagnosis.

There have been enormous efforts in this origin-tracing deduction within different multiple primary tumors, especially multifocal thyroid cancer, and prostate cancer [3, 4]. The majority of the studies have focused on the metastasis model. Clonality analysis of synchronous GCA and distal gastric cancer revealed potential benefit of immunotherapeutic treatments [5]. However, multicentric origin model of MECC still lacks theoretical support. To comprehend the associations and bolster the therapeutic efficacy of treatments, it is imperative to delve into the origins and molecular mechanisms underlying the progression of MECC. To address this question, we performed whole-genome sequencing (WGS) of 20 tumor foci and paired normal samples from 10 MECC patients to investigate the clonal origin and the genome characteristics of MECC.

## Materials and methods

### Sample collection

We extracted statistical data on MECC cases from patients with EC or GCA collected from the Institute of Clinical Pathology, Shantou University Medical College between 1999 and 2017. Sequencing samples were collected from patients undergoing resection at the Cancer Hospital of Shantou University Medical College from February 2014 to January 2017. All patients underwent surgery without receiving any chemotherapy or radiation prior to surgery. This study was conducted with the approval of the ethics committee of Shantou University Medical College. All individuals confirmed the ethical approval by signing the informed consent form. The study was performed by the Declaration of Helsinki. We obtained two separate tumor foci from 10 individuals, with each specimen being at least 0.5 cm away from the other one.

### DNA extraction and whole-genome sequencing

A total of 20 freshly frozen tumor samples from 10 individuals with MECC were cut into sections of 20 μm and alternate sections were taken for DNA extraction. The paired normal esophageal tissues or paired blood DNA were used as controls. Manual microdissection was performed using a 1 mL syringe needle and the tumor purity > 80%. WGS was performed on an Illumina HiSeq X-ten platform.

### Data analysis

The paired-end clean reads were aligned to the human reference genome (UCSC GRCh37/hg19) using the Burrows-Wheeler Aligner (BWA) (v0.1.22) [6]. Alignments were then filtered for duplicate reads using SAMBLASTER (v0.4.7) [7], BAM files were indel realigned and base quality scores were recalibrated according to Genome Analysis Toolkit (GATK) best practice [8]. SAMtools (v1.0) [9] was utilized to identify SNPs and indels. MuTect (v1.1.4) identified candidate somatic mutations by Bayesian statistical analysis, and a minimum of 10 reads both in the matched non-normal and normal samples were required to declare that a site was adequately covered for mutation calling [10]. Small somatic indels were performed by Strelka tools (v1.0.13) [11]. The filtered variants were functionally annotated by ANNOVAR (v2013-08-23) [12] using the RefGene database dbSNP142 [13], 1000 Genomes Project [14], SIFT [15], PolyPhen [16], GO [17] and KEGG [18].

### Detection of germline mutated genes

Mutations detected in normal tissues were compared with the CGC (Cancer Gene Census) database to screen for possible cancer susceptibility genes. The mutations were filtered as follows: (i) mutation sites less than 10× depth were filtered out, (ii) High-frequency SNP sites mostly represent common polymorphisms, which are widely present in different populations and usually not associated with diseases. Therefore, SNP sites with an allele frequency greater than 0.001 in dbSNP142, the 1000 Genomes Project database, and the complete Exome Aggregation Consortium (ExAC) database were filtered out. However, we retained variants from the COSMIC database because it specializes in collecting and recording cancer-related mutations, which have significant clinical and biological importance. (iii) intergenic regions, noncoding regions and intron regions and synonymous mutations were filtered out, (iv) mutations in the genome repeat regions were filtered out.

### Somatic variation analysis

The overlap of somatic SNV calls between matched tumor samples was filtered as follows: (i) For positions in one sample with high-quality alternative allele reads, if there was a single read containing the alternative allele in the paired sample (retrieved from bam files), the positions were considered to be shared SNVs. (ii) SNVs were considered unique if the corresponding matching sample contained only reference bases covering the position. (iii) For unique SNVs that fell in regions of LOH in the paired sample, they were filtered when performing the clonal and phylogenetic analyses, as one cannot determine whether these SNVs were truly unique to the sample or had been lost in the other sample [19]. The copy number variations (CNVs) were identified using Control-FREEC (v6.7) with tumor and paired normal SAM pileup and dbSNP files as input. GISTIC2.0 [20] was utilized to evaluate the reproducibility and significantly aberrant regions of CNVs. Validation of a set of nonsynonymous mutations randomly selected via Sanger sequencing yielded an average validation rate of 94.34% (Table S1).

### Clone analysis

Pyclone (version 0.12.7) was used to evaluate the clonal population structures. Structural variation (SV) was identified using Crest (v0.0.1), a software tool that uses soft-clipped reads to directly map the breakpoints of structural variations.

### Analysis of CNV and LOH

Control-FREEC constructed, normalized, segmented, and analyzed copy number and B-allele frequency (BAF) profiles to assign genotype status to each genomic region. CNVs and LOH regions were annotated with read count, copy number, BAF, and genotype information for each window [21].

### Prediction for neoantigens

The HLA alleles were predicted using polysolver [22] and the mutant and wild peptide binding affinity were calculated by NetMHCpan [23]. Candidate neoantigens were identified as those with a predicted mutant peptide binding affinity of < 500 nmol/L and less than wild peptide binding affinity.

### Statistical analysis

The potential factors associated with the detection rate of multifocal esophageal squamous cell carcinomas (MECCs), including age and sex, were analyzed using logistic regression analysis, calculating hazard ratios (HR), and determining the relative 95% confidence intervals (CIs). To identify independent prognostic factors (age, sex, and tumor state), all significant variables on univariate Cox regression analysis (*p* ≤ 0.05) were subjected to multivariate Cox regression analysis. Statistical analyses were performed using R (version 4.2.1). Tests were two-sided and unpaired, and the significance threshold was set at *p* < 0.05.

## Results

### Clinical sample type of MECC

**Fig. 1:**
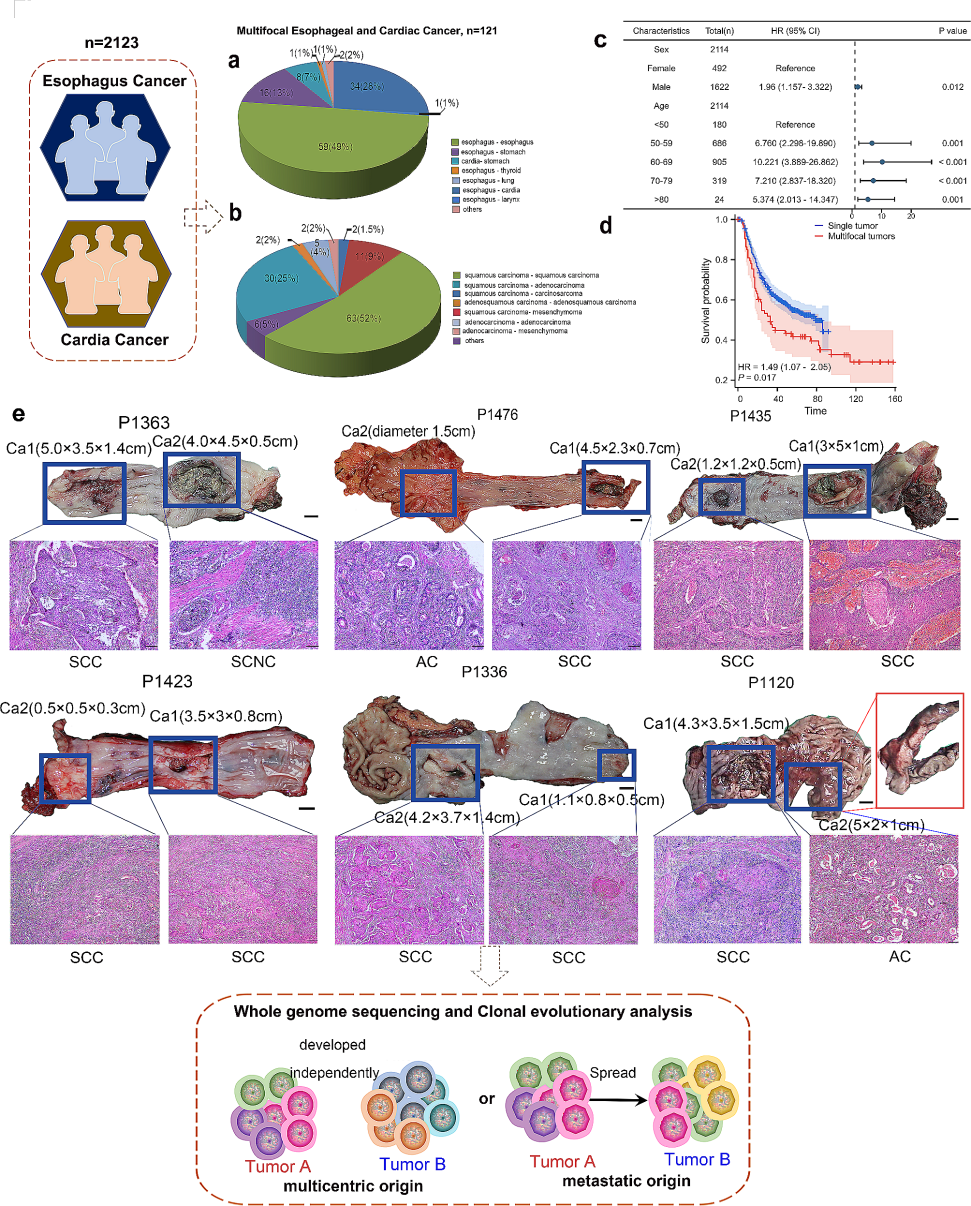
Clinical description of MECC. **a**-**b.** Lesion locations (**a**) and pathological types (**b**) of MECC in the Chaoshan area, China. **c.** The forest plot of multivariate logistic regression analysis. The length of the horizontal line represents the 95% CI for each group. Participants with HR > 1 exhibited a higher risk of MECC. CI, confidence interval. **d.** Survival analysis showing that MECC was associated with poor overall survival (OS). **e.** Resected MECC specimen (scale bars, 1 cm) and hematoxylin and eosin (H&E) staining (SCC-squamous cell carcinoma; AC-adenomatous carcinoma; SCNC- small cell neuroendocrine carcinoma; scale bars, 50 μm)

MECC is commonly found in the esophagus and gastroesophageal junction of patients with esophageal cancer (EC) and gastric cardia adenocarcinoma (GCA) in the Chaoshan area, where the incidence of EC and GCA is high. In this study, we collected clinical data of EC and GCA patients from 1999 to 2017 to investigate the clinical characteristics of MECC. Out of 2123 cases, MECC was detected in 121 cases (5.65%). The predominant locations for MECC were esophagus-esophagus (49%), esophagus-cardia (28%), and esophagus-stomach (13%) (Fig. 1a). The main pathological patterns observed were squamous cell carcinoma-squamous cell carcinoma (51%), squamous cell carcinoma-adenocarcinoma (27%), and squamous cell carcinoma-gastrointestinal stromal tumor (9%) (Fig. 1b). Interestingly, the preferred location of MECC was consistent with the high incidence rates of EC and GCA in the Chaoshan region, implying EC and CGA may share a similar carcinogenic cause or pathogenesis [2].

Then we identified the factors associated with a higher detection rate of MECC based on demographic data. The major risk factor for MECC was age and the risk of MECC increased with age, especially in age between 60 and 69 years (HR = 10.221, *p* < 0.001). Men were much more likely to develop from MECC than women (HR = 1.96, *p* = 0.012) (Fig. 1c). We also conducted prognostic analysis on a cohort of 541 EC and GCA patients with available follow-up data (MECC patients, *n* = 74; single tumor patients, *n* = 467) by Cox regression analysis. MECC patients showed worse prognosis compared with single tumor patients (*p* = 0.017) (Fig. 1d). Prognostic factors with *p* < 0.05 in the univariate analysis were included in the multivariate analysis (Table 1). The results showed man sex and MECC were associated with poor prognosis (HR = 1.374, *p* = 0.040; HR = 1.408, *p* = 0.041).

**Table 1:** Prognostic factors for overall survival of patients with EC and GAC

Characteristics	Total(*N*)	Univariate analysis			Multivariate analysis	
		HR(95% CI)	*P* value		HR(95% CI)	*P* value
**Sex**	541					
Female	141	Reference			Reference	
Male	400	1.428 (1.058–1.928)	0.020		1.374 (1.014–1.860)	0.040
**Age**	541	1.004 (0.989–1.018)	0.627			
< 50	43	Reference				
50–60	182	0.998 (0.618–1.612)	0.995			
60–70	225	1.004 (0.629–1.602)	0.986			
70–80	85	1.030 (0.609–1.740)	0.913			
> 80	6	0.788 (0.234–2.650)	0.7			
**Tumor state**	541					
Single tumor	467	Reference			Reference	
Multifocal tumors	74	1.485 (1.074–2.055)	0.017		1.408 (1.015–1.955)	0.041

**Table 2:** Clinical-pathological parameter of 6 MECC patients

Patient ID	Tumor ID	Tumor location	Gross type	Histological subtype	Grade	pT-stage	*N*-stage	Gender	Age
P1120	Ca1	Lower of Esophgus	Ulcerative type	SCC	G2	T3	N3	Male	58
	Ca2	Gastric Cardia	Infiltrating type	AC	G3	T3			
P1336	Ca1	Upper of Esophgus	Medullary type	SCC	G2	T1	N1b	Male	50
	Ca2	Lower of Esophgus	Ulcerative type	SCC	G2	T3			
P1363	Ca1	Upper of Esophgus	Medullary type	SCC	G2	T3	N3	Male	51
	Ca2	Middle of Esophgus	Ulcerative type	SCNC	G3	T2			
P1423	Ca1	Middle of Esophgus	Medullary type	SCC	G1	T3	N2	Male	60
	Ca2	Lower of Esophgus	Ulcerative type	SCC	G1	T1			
P1435	Ca1	Upper of Esophgus	Ulcerative type	SCC	G2	T3	N2	Male	62
	Ca2	Lower of Esophgus	Fungating type	SCC	G2	T1			
P1476	Ca1	Upper of Esophgus	Ulcerative type	SCC	G2	T3	N0	Male	62
	Ca2	Gastric Cardia	Ulcerative type	AC	G3	T1			

Squamous cell carcinoma (SCC); Adenocarcinoma (AC); Neuroendocrine small cell carcinoma (SCNC); Adenosquamous carcinoma (ASC)

### Clonal architecture of MECC

**Fig. 2:**
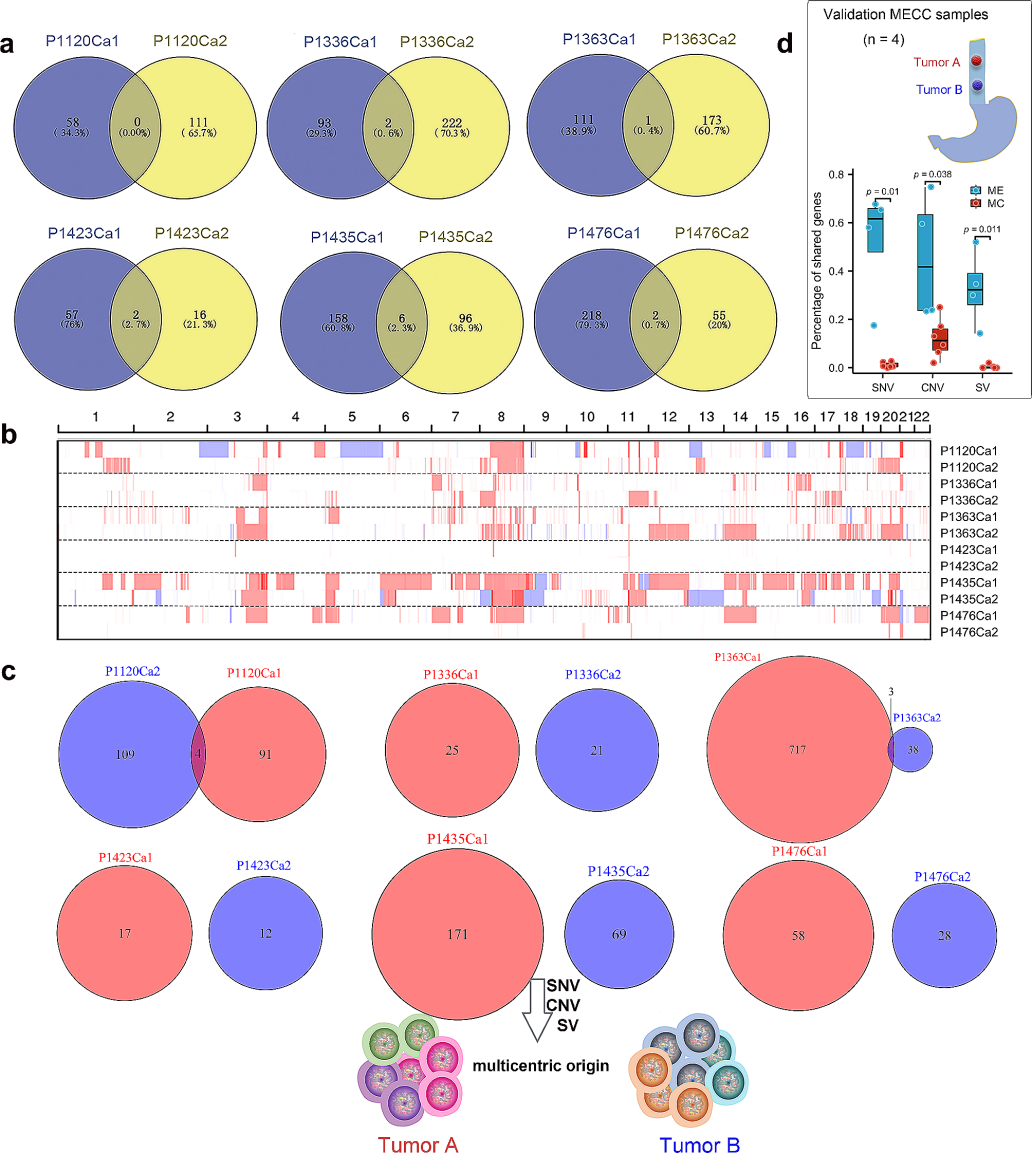
Genetic heterogeneity and clonal relationship of MECC. **(a)** The total number of shared somatic exonic mutations between different subjects is shown in Venn diagrams. **(b)** Copy number alterations heatmaps of MECC. **(c)** The Venn diagrams display the number of shared structural variation sites for each subject. **(d)** The proportion of shared SNV sites, CNV genes and SV sites between two groups (Wilcoxon rank sum test)

WGS was performed on genomic DNA from 10 MECC patients to determine the clonal relationship between MECC foci. In total, 20 tumor samples and 10 matched normal samples were sequenced, with an average depth of 50× and 30× for tumor and normal samples, respectively. We compared their genomic profiles for shared and individual alterations within each patient. Six patients were identified as following the multicentric origin (MC) model, comprising 4 cases with multifocal EC and 2 cases with EC-GCA (Fig. 1e). The clinical and pathological parameters of these patients are shown in Table 2. The degree of shared somatic nonsynonymous SNVs and indels varied from 0 to 2.7% among patients, indicating a genetically independent multicentric origin (Fig. 2a). Then we compared the CNV spectrum within each patient. The majority of CNV regions were detected in only one tumor focus (Fig. 2b). Most of the cases (expect for P1423) tended to harbor majority of individual-specific CNVs in paired tumor foci (0.2–17.0%). P1423 had 25.0% shared CNV genes due to few CNV events were detected in Ca2.

Additionally, we analyzed the structural variations (SV) in MC patients (Fig. 2c), revealing a distinct SV spectrum between paired tumor foci. The percentage of shared SV events in the MC model ranged from 0 to 0.04%. Taken together, our results demonstrated pronounced variable extents of heterogeneity between foci from the same patient, and confirmed 6 cases (P1120, P1336, P1363, P1423, P1435, and P1476) were MC model.

To gain further insights into the clonal origin types, the remaining 4 cases were used as the validation group, comprising a total of eight esophageal tumor foci (Fig. 2d). The paired foci of the validation group exhibited a significant amount of overlap in variations, indicating that the validation group follows a metastatic origin model (ME). The ME cases showed a high shared mutation rate (ranging from 58 to 67.6%), which was significantly higher than the MC group (*p* = 0.022). Moreover, the ME group had a higher number of overlap CNV genes and SV sites compared to the MC group (*p* = 0.038 and 0.011).

### Increased germline mutation in immune genes in MC model

**Fig. 3:**
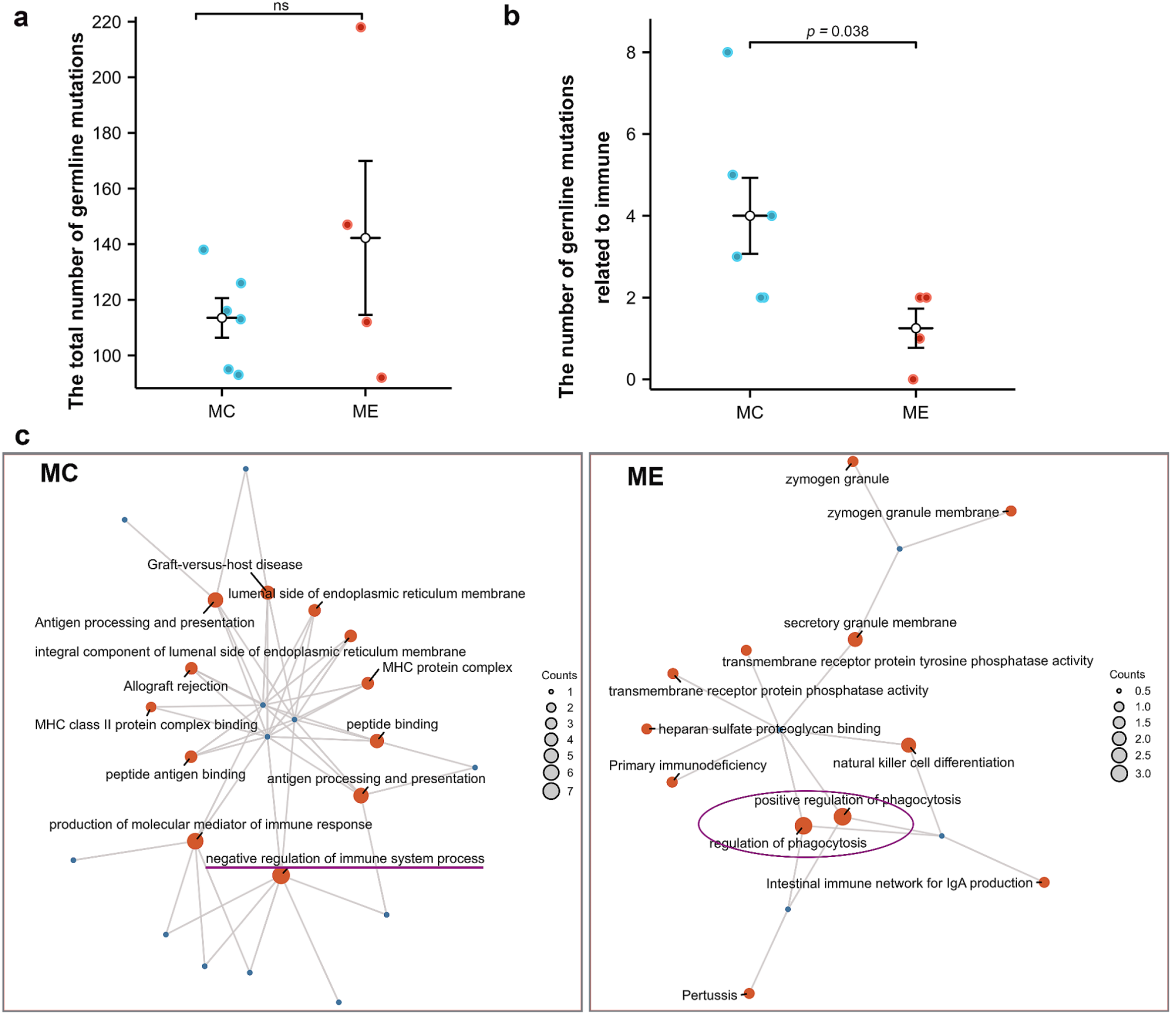
Germline mutation comparison of MECC. **a-b.** Comparison of the total number of germline SNVs and the number of germline SNVs related to immune between MECC-MC and MECC-ME samples (nonparametric test, two-sided). **c.** GO enrichment networks of germline SNVs related to immune response in MECC-MC and MECC-ME

Germline mutations also play a role in the mechanism of tumorigenesis. We compared the germline mutation status of the ME and MC groups. The rare variants were selected, and the functions of genes were annotated by the Kyoto Encyclopedia of Genes and Genomes (KEGG) and Gene Ontology (GO). Overall, we found no significant differences in the total number of germline mutations between the two groups (Fig. 3a). However, when we focused on germline mutations involved in tumorigenesis-related processes such as cell cycle regulation, cell proliferation, DNA repair, cell adhesion, and immune response, we discovered that rare germline mutations associated with immune were significantly more prevalent in MC cases than in ME cases (*p* = 0.038) (Fig. 3b).

We further conducted enrichment analysis of immune genes. In MC cases, immune genes were enriched in functions related to the regulation of the immune system and antigen processing. On the other hand, immune genes in ME cases were enriched in functions related to the regulation of phagocytosis and transmembrane receptor protein phosphatase (Fig. 3c). We hypothesized that inherited immune system defect may contribute to the tumorigenesis of the MC model. Further studies involving a larger number of cases is required to confirm the findings.

### The evolution of MC cases

**Fig. 4:**
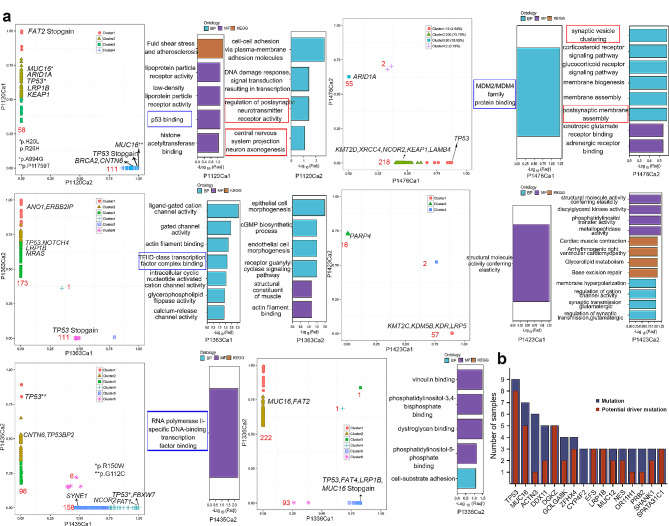
Characteristics of MECC-MC. **(a)** Clonal frequency comparison of SNVs detected in MECC-MC cases (left panel). The number of SNV mutations in each clone cluster was calculated.The red numbers indicate the number of nonsynonymous mutations. Key mutations determined by clonal frequency analysis and deleterious prediction (a SIFT score ≤ 0.05 or a PolyPhen-2 score ≥ 0.957) are marked. The somatic mutations for each tumor focus were subjected to enrichment analysis for GO terms and KEGG pathways (right panel). Mutations in P1336Ca2 and P1435Ca1 did not show enrichment in the results. **(b)** The stacked bar chart represented the number of samples with mutated genes and potential driver mutation genes

During tumor evolution, some mutations may be early events that arise in the common ancestral cells at the initiation stage of the tumor. As the tumor evolves and expands, these early mutations are transmitted to more cells, leading to an increase in clone frequency. We investigated the genomic evolution process of tumor foci by performing clonal frequency analysis. The variations in majority of tumor foci exhibited multiple clonal clusters, indicating a multiclonal formation pattern. However, in P1120 Ca2, P1423 Ca1 and Ca2, all of them had a single clone, suggesting that they were of monoclonal origin. The deleterious mutations with high clonal frequencies (**≥** 50%) indicated that key mutations may give rise to tumors potentially be driver genes (Fig. 4a). The mutation sites of potential driver genes, such as those in *TP53*, *FAT2*, *EGFR*, *BRCA2*, and *APC*, could be detected only in some lesions, indicating that different lesions of multicentric MECC individuals might undergo independent clonal expansion and become transformed as separate clones. However, *TP53*, *MUC16* and *DGKZ* were identified as potential driver mutation genes in 8/9, 5/7 and 5/5 samples, respectively, indicating that despite the independent clonal origins, there are similarities in the biological events experienced within the tumors (Fig. 4b**)**.

So, we performed enrichment functional analysis of non-silent mutations and found *TP53* related binding terms were enriched in 4/7 SCC samples (blue boxes in Fig. 4a) and neurological terms were enriched in 2/2 GAC samples (red boxes in Fig. 4a). We paid particular attention to P1363 because two histology types were observed: SCC (Ca1) and SCNC (Ca2) which was very rare in the clinic. The histology of P1363Ca2 was confirmed by immunohistochemical staining (Supplementary Fig. 1). Clonal frequency analysis revealed distinct subclones in each tumor focus, with two clusters in Ca1 and three clusters in Ca2. The mutations related to each subclone were enriched in different GO terms. Ca1 exhibited anomalies in channel activity, TFIID-class transcription factor complex binding, and respiratory chain complex III, while Ca2 showed enrichment in mutations related to epithelial cell morphogenesis and endothelial cell morphogenesis. Interestingly, they both shared a common term: actin filament binding.

**Fig. 5:**
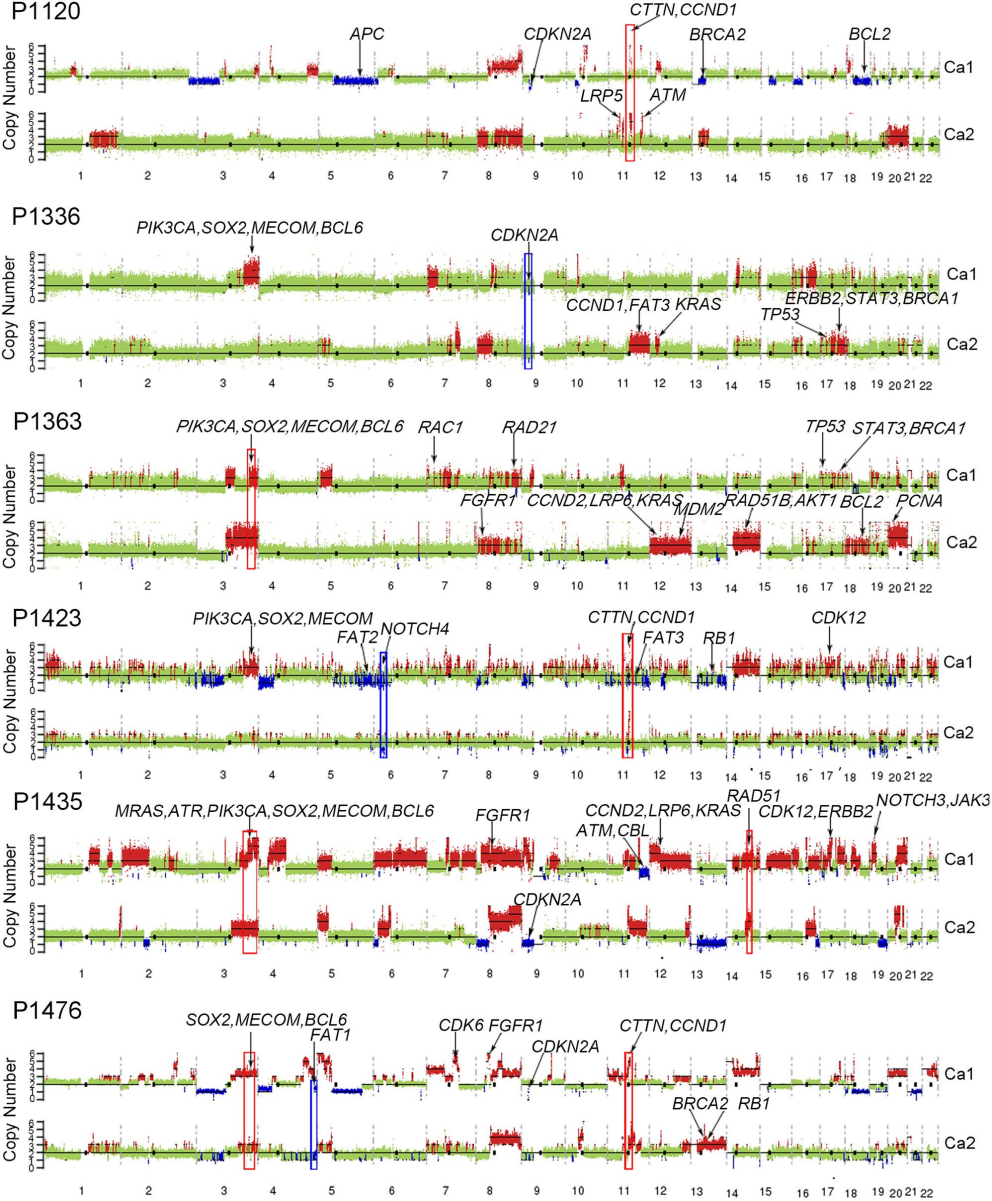
Copy number alteration profiles across chromosomes. The red and blue peaks show the locations with copy number amplification and copy number deletion, respectively. Cancer genes and the high frequency-altered genes are labeled. Boxes indicate the overlapping alterations between samples

Although the paired tumor foci of MECC-MC cases exhibited distinct mutation sites, they showed shared CNV regions (Fig. 5). For instance, amplification of 3q26 was detected in paired tumor foci of 50% cases (P1363, 1435 and P1476), harboring oncogene *PIK3CA*, *SOX2* and *BCL6*. Additionally, amplification of *CCND1* was detected in paired tumor foci of P1120, P1423, and P1476. Deletion of *CDKN2A* was detected in paired tumor foci of P1336. Hence, CNVs may serve as potential targets for treatment of MECC-MC.

### Detection and therapeutic targets of MECC

**Fig. 6:**
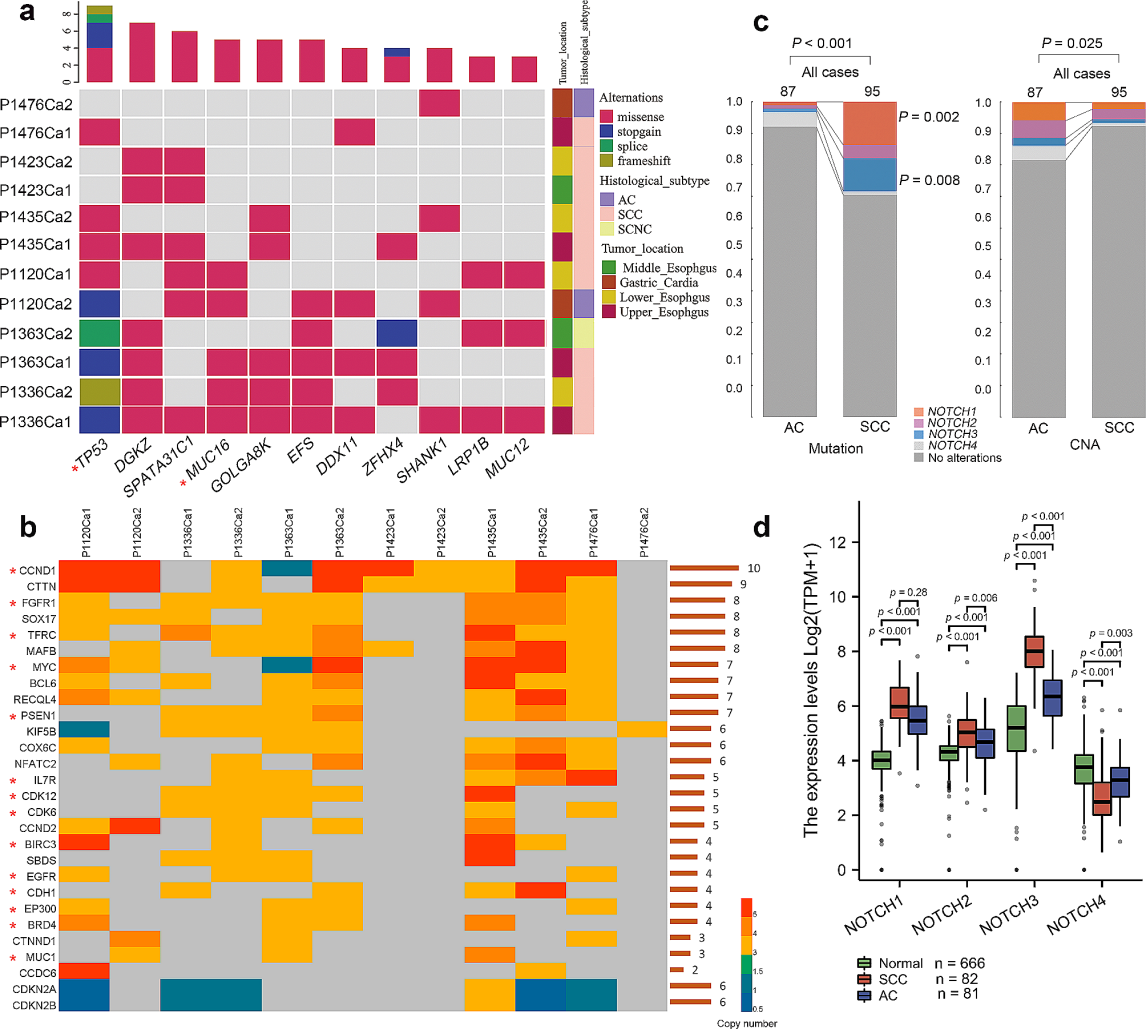
Detection and therapeutic targets of MECC. **(a)** The frequency of neoantigen. Binding affinity of the neoantigen for MHC was predicted across all 9–11 amino acid peptides generated from non-silent mutations and the corresponding wild-type peptides using NetMHCpan algorithms. The predicted binding affinity of < 500 nmol/L were selected. The target genes with clinically available targeted drugs were marked with *. **(b)** The heatmap of CNV gene with high frequency. The bars on the right represent the number of samples with CNV occurrences. The target genes with clinically available targeted drugs were marked with *. **(c)** Comparison of the *NOTCH* family variation from public database (AC, *n* = 87; SCC, *n* = 95). *P*-values were computed by nonparametric test. **d.** *NOTCH* family mRNA expression levels comparison in SCC, AC, and normal samples. *P*-values were computed by Kruskal-Wallis test and Dunn’s test

Neoantigens arise as a consequence of tumor-specific mutations. Polysolver and NetMHCpan were used to predict the neoantigens affinity for major histocompatibility complex (MHC) that could be targets for clinical immunotherapy (Supplementary Fig. 2). The mutations predicted as neoantigens in more than 2 tumor foci were shown in Fig. 6a. All the mutated genes were identified as potential driver genes during evolutionary analysis. Upon querying the Therapeutic Target Database [24], it was found that *TP53* and *MUC16* are targets for targeted therapy, and there are clinically available targeted drugs.

Compared to the heterogeneity of mutations, the degree of shared copy number variations is more suitable as therapeutic targets. A total of 28 genes showed CNVs in more than 2 tumor foci (Fig. 6b). For example, *CCND1* and *CTTN* showed amplification in more than 9 tumor foci, while *CDKN2A* and *CDKN2B* exhibited deletion in more than 6 tumor foci. Simultaneously, we also observed that, compared to mutated genes, more genes with CNA have targeted therapeutic drugs available clinically.

As mentioned above, 25% of the cases with MECC had a histological subtype of squamous carcinoma-adenocarcinoma. Therefore, we further analyzed the similarities and differences between SCC and adenocarcinoma (AC). We found CNVs of genes in the Notch signaling pathway were common in both SCC and AC. So we further analyzed the Notch family (*NOTCH1*-*4*) variation data from esophageal carcinoma TCGA Pan-Cancer (AC, *n* = 87; SCC, *n* = 95) [25]. The number of mutated *NOTCH1*, and *NOTCH3* in SCC was significantly higher than AC (*P* = 0.002 and 0.008). However, AC had more CNVs in the notch family than SCC (*P* = 0.025) (Fig. 6c). We further analyzed the mRNA expression of NOTCH1-4 in SCC and AC (Fig. 6d**)**. The expression levels of NOTCH1, NOTCH2, and NOTCH3 were significantly higher in both SCC and AC compared to normal samples (*p* < 0.001). NOTCH1, NOTCH2, and NOTCH3 exhibited higher expression in ESCC compared to EAC, with significant differences observed for NOTCH2 (*p* = 0.006) and NOTCH3 (*p* < 0.001). Additionally, the expression of NOTCH4 in both SCC and AC was significantly lower than in normal samples (*p* < 0.001), and ESCC showed a notably lower expression compared to EAC (*p* = 0.003). Therefore, in the treatment and detection of multifocal cancers involving SCC and AC, the differences in the Notch family should also be taken into consideration.

## Discussion

In this study, we found that multifocal esophageal and cardiac cancer is associated with worse survival compared with single tumor, which suggest MECC cases should be intensively studied. The genomic analyses lead us to investigate the clonal origin of multifocal esophageal and cardiac cancer. First, we demonstrated that multifocal esophageal and cardiac cancer can be divided into either having a metastatic origin or a multicentric origin, which suggests that MECC cannot be considered as single cancer for clinical consideration and treatment even the tumor foci are in the same pathological type. Sequencing data can reveal the relationship between tumor foci and guide the target therapy and early cancer detection.

Both environmental factors and genetic predisposition underlie the risk of cancer. Therefore, we compared the germline mutations between MECC-MC and MECC-ME to observe the influence of genetic susceptibility in multiple cancers. Interestingly, MECC-MC patients harbored more germline alterations in immune mechanisms. In our previous studies, a background investigation in the Chaoshan area showed 68.85% of chronic inflammation in high-risk populations for EC and which may play an important role in the high incidence of EC/GCA [26]. Microbiota stimulation, smoking, drinking, or other factors can cause chronic inflammation and induce immune response of the digestive tract microenvironment [27–30]. A compromised immune response can potentially trigger carcinogenesis at various focal points, leading to the independent development of primary cancers. Also, we need more MUGC cases to exploit that defects in immune are associated with the risk for tumor foci of multicentric origin.

For somatic mutation, we found *TP53* related binding terms are enriched. Consistent with previous studies, TP53 is one of the most frequently mutated genes which occur in approximately 50–80% of ESCC cases [31, 32]. Mutated TP53 often result in the loss of tumor suppressor functions, such as DNA repair, cell cycle regulation and apoptosis [33]. Mutant p53 proteins can promote cancer cell survival and tumor progression by functioning as homeostatic factors that detect and shield cancer cells from stress stimuli related to transformation [34]. These stimuli include the immune system, oxidative and proteotoxic stress, metabolic imbalance, interactions with the tumor microenvironment, and DNA lesions.

As most of MECC-MC can be divided into squamous carcinoma and adenocarcinoma, we found the Notch signaling pathway the main variation of SCC was mutation [35], and CNVs were more frequent in AC. These data imply that the Notch signaling pathway participates in the tumorigenic process through different paths in different pathologic types of cancer. As the evolution of multiple tumors had relative independence, most of the potential driver genes harbor different mutational sites, indicating next-generation sequencing can serve as an effective method for clinically distinguishing the origins of multifocal cancers. Considering that whole-genome sequencing is still not widely applicable for clinical testing on a large scale, the high-frequency driver genes (for example, *TP53*, *MUC16* and *DGKZ*) from multiple lesions could be selected to establish a gene-targeted sequencing panel for distinguishing origin types of multiple tumors, thereby guiding clinical treatment. Compared to unique mutation sites, MECC-MC showed several shared CNV regions harbored oncogenes or tumor suppressor genes. Thus, CNVs had higher clinical targeted therapy value for MECC-MC cases. For example, the amplification of FGFR1 were detected in the paired tumors of three cases, which could be treat with target drugs [36].

The limitation of our current study lacks a more in-depth exploration of the mechanisms and etiology of multifocal cancer formation. Furthermore, future studies with larger cohorts are necessary to validate our conclusions and explore the broader applicability of our findings.

## Conclusions

WGS deciphers the clonal origin of multifocal cancer. The extent of inter-tumor heterogeneity suggests two types of clonal origin of MECC. This dynamic clonal evolution will provide both a theoretical and translational basis for identifying new targets and designing cancer precision medicine strategies.

### Electronic supplementary material

Below is the link to the electronic supplementary material.

Supplementary Material 1

Supplementary Material 2

## Data Availability

The datasets supporting the conclusions of this article are available within the article and its supplementary files. The sequencing raw data are available via the corresponding author upon reasonable request.
