# Supplementary material for: Genomic characteristics and evolution of Multicentric Esophageal and gastric Cardiac Cancer
Source: Biol Direct. 2024 Jul 1;19:51. doi: 10.1186/s13062-024-00493-y (PMC11218177; doi:10.1186/s13062-024-00493-y)
Supplement: Supplementary file 2 — Supplementary Material 2 [file 13062_2024_493_MOESM2_ESM.pdf]

Genomic Characteristics and Evolution of Multicentric Esophageal and Gastric Cardiac Cancer

Correspondence: Min Su, E-mail: minsu@stu.edu.cn

Institute of Clinical Pathology & Department of Pathology, Shantou University Medical College, Shantou 515041, Guangdong, China.

Supplementary Figure S1

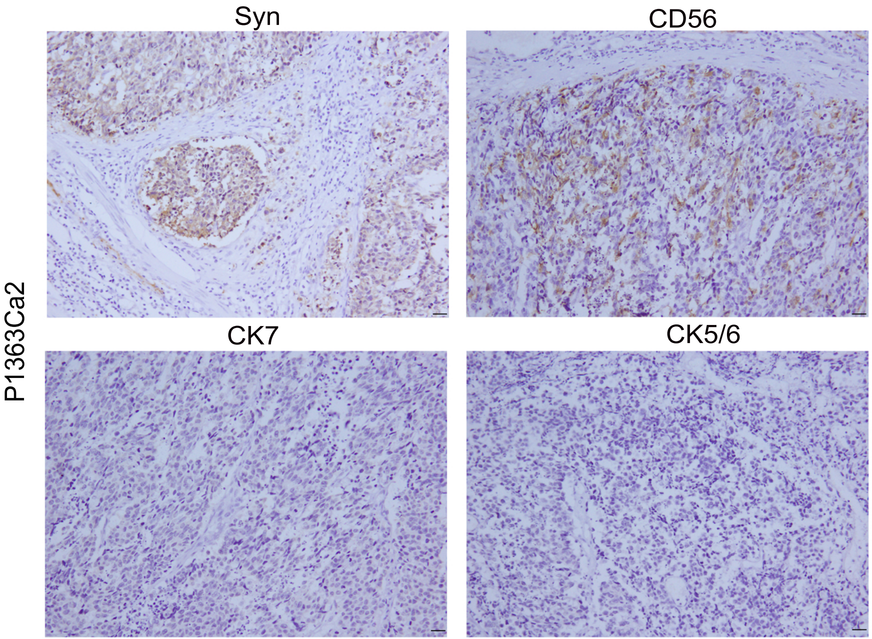

Supplementary Figure S1.Immunohistochemical staining of P1363 Ca2 (scale bars, 50μm).

Supplementary Figure S2

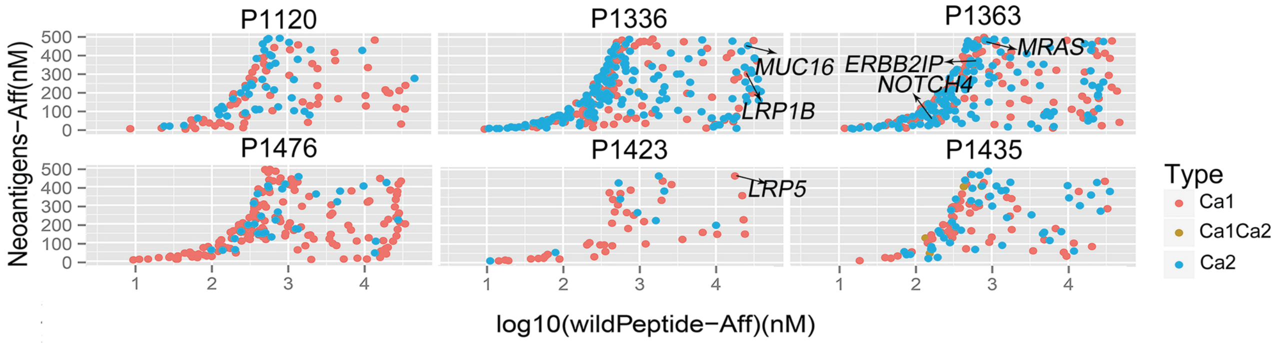

Supplementary Figure S2. Binding affinity of the neoantigen for MHC was predicted from non-silent mutations and the corresponding wild-type peptides using NetMHCpan algorithms. Mutated peptides with predicted binding affinity of <500 nmol/L were plotted and key mutations were labeled. The lower affinity constant indicated stronger affinity.
